# Use of Twitter Amplifiers by Medical Professionals to Combat Misinformation During the COVID-19 Pandemic

**DOI:** 10.2196/38324

**Published:** 2022-07-22

**Authors:** Regina Royan, Tricia Rae Pendergrast, Marina Del Rios, Shannon M Rotolo, N Seth Trueger, Eve Bloomgarden, Deanna Behrens, Shikha Jain, Vineet M Arora

**Affiliations:** 1 Department of Emergency Medicine Northwestern University Chicago, IL United States; 2 Feinberg School of Medicine Northwestern University Chicago, IL United States; 3 Department of Emergency Medicine Roy J and Lucille A Carver College of Medicine University of Iowa Iowa City, IA United States; 4 Department of Pharmacy University of Chicago Medicine Chicago, IL United States; 5 JAMA Network Open Chicago, IL United States; 6 Division of Endocrinology Northshore University Health System Evanston, IL United States; 7 Department of Pediatric Critical Care Medicine Advocate Children’s Hospital Park Ridge, IL United States; 8 Division of Hematology and Oncology University of Illinois Chicago, IL United States; 9 Department of Medicine Pritzker School of Medicine University of Chicago Chicago, IL United States

**Keywords:** social media, combating disinformation, misinformation, infodemic, amplifier, COVID-19, advocacy, public health communication, disinformation, medical information, health professional amplifier, healthcare profession, health care profession, Twitter, public communication, health information, health promotion

## Abstract

Social media is an important tool for disseminating accurate medical information and combating misinformation (ie, the spreading of false or inaccurate information) and disinformation (ie, spreading misinformation with the intent to deceive). The prolific rise of inaccurate information during a global pandemic is a pressing public health concern. In response to this phenomenon, health professional amplifiers such as IMPACT (Illinois Medical Professional Action Collaborative Team) have been created as a coordinated response to enhance public communication and advocacy around the COVID-19 pandemic.

## Introduction

In the era of global pandemics, the prolific rise of medical misinformation is a pressing public health concern. The Surgeon General of the United States issued the following directive in 2021: “Health misinformation is a serious threat to public health. It can cause confusion, sow mistrust, harm people’s health, and undermine public health efforts. Limiting the spread of health misinformation is a moral and civic imperative that will require a whole-of-society effort” [1]. In early 2019, the American Medical Association issued a letter to the chief executive officers of the country’s 6 leading social media and technology companies urging them to ensure their users have access to accurate, timely, and scientifically sound information on vaccines [2]. Despite this call in 2019, many public health professionals found themselves woefully underprepared to face the deluge of misinformation surrounding COVID-19 and the subsequent vaccine rollout [3]. As an organic response to this phenomenon, a new type of professional organization was born: the health professional amplifier.

IMPACT (Illinois Medical Professional Action Collaborative Team) is a 501(c)(3) nonprofit organization designed to help physicians and health professionals engage in grassroots networks, advocate for evidence-based solutions, advise influential stakeholders, and amplify solutions to protect individuals and communities across the state. IMPACT’s Twitter account, @IMPACT4HC, is a verified account with 3232 followers.

Twitter has the option to create a private group direct messaging thread, allowing up to 50 individuals to be included in a single messaging conversation. To amplify the message of the @IMPACT4HC account, IMPACT founders (authors VMA, SJ, and EB) created a private group messaging thread of key members across specialties, professions, areas of expertise, and racial and ethnic backgrounds, thus creating an “IMPACT amplifier.” The IMPACT amplifier group connects 39 accounts, 7 of which are verified accounts, with over 320,000 followers collectively.

**Figure 1:**
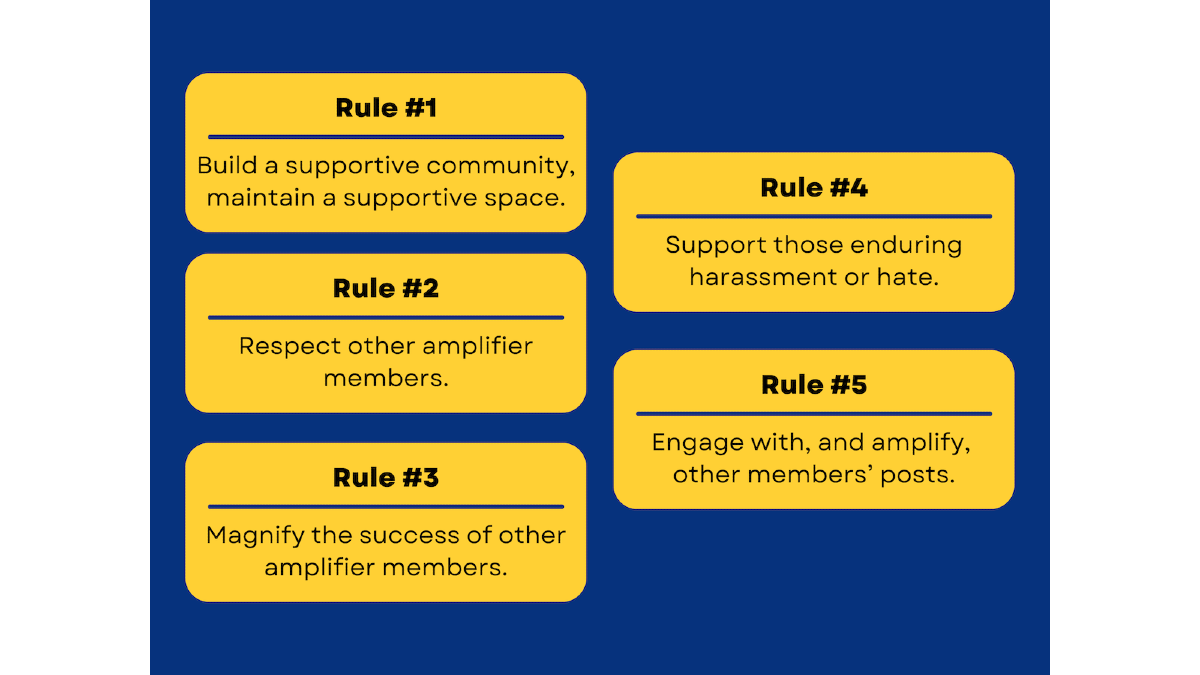
Rules of engagement for Twitter amplifiers.

We define an amplifier as a private group of people with a similar mission, who use Twitter for private discussion, brainstorming, and planning, and the subsequent public communication and promotion of the group’s work and messaging (Figure 1). This back-channel discussion allows for collaboration between a group of people with similar goals, missions, or agendas, who otherwise may not have open communication channels or perhaps have not even met in person. The goals of an amplifier are to connect people, collaborate, strategize, and amplify ideas on Twitter and other social media platforms.

Since its creation at the start of the pandemic, IMPACT members have used this amplifier to share IMPACT posts and infographics on social media to communicate quickly about pertinent pandemic-related questions and concerns and to stay on top of rapidly changing information across the state. This group of Illinois-based medical professionals, science communication experts, and researchers within IMPACT have used the IMPACT amplifier to facilitate interdisciplinary discussion and coordinate action [4-6]. In this paper, we will discuss this work within the following broad themes: rapid dissemination and promotion of accurate medical information and public health guidance, combating disinformation, and countering harassment.

## Promotion of Timely and Accurate Medical Information

The volume of available health information is both massive and growing; the number of publications in scientific papers is increasing at a rate of over 8% per year, and greater than 1 million new publications are listed in PubMed annually [7]. This amount of information can be overwhelming to both health professionals as well as the lay public and press. The pandemic accelerated the amount of published scientific health information in an unprecedented way. At the same time, the general public needed access to this information rapidly. This deluge of information in the setting of an evolving global pandemic created what the World Health Organization dubbed as an “infodemic” [3]. Scientific claims were being shared without an appreciation of the nuances and limitations of the scientific process, and many incorrect conclusions were shared as fact, in various degrees of good and bad faith [8]. Throughout the pandemic, science communication and public health messaging failed to live up to their potential. The public was often left to decipher confusing and sometimes contradictory information from politicians, agency officials, and an abundance of “armchair epidemiologists,” which left many questioning previously trusted sources of public health guidance. The need for efficient, real-time communication strategies among frontline health care workers, medical professionals, science communicators, and public health experts led to the formation of health communication amplifiers. These amplifiers provided an opportunity to work together to curate content on social media by filtering, interpreting, and amplifying.

First, health professionals can work via amplifiers to select and highlight high-impact information, directing attention to the most relevant studies and data to promote while both implicitly and explicitly prioritizing topics to address and respond to. Next, health professional amplifiers can use their individual and group knowledge, experience, and platform to interpret data, clarify, and explain high-impact research, or to explain why certain findings may not be relevant (eg, clarifying the difference between correlation and causation in observational studies). Importantly, these amplifiers can correct misinformation by calling out incorrect interpretations of research and flawed research methods. Third, health professionals can distill complex information into valuable takeaways (eg, infographics, videos) and distribute them more broadly via an amplifier. Health professionals can also partner with community groups and other local organizations to prevent and address health misinformation [9]. This becomes especially important when engaging with disenfranchised racial and ethnic groups who are often the target of misinformation campaigns or who may not have easy access to culturally relevant and language-concordant reputable sources [10,11]. While many of these objectives can be worked on by individuals, amplifiers can help health professionals find and interpret new data, workshop communication strategies, and share (ie, amplify) and lend credibility to each other’s work.

Additionally, many journalists in traditional media use or monitor social media, and amplifiers can help integrate messages and trusted messengers into larger, traditional media opportunities. Health professional amplifiers can also provide a window into the unique perspectives of frontline health care providers. Particularly in the early part of the pandemic, much of the public was sheltered from the suffering and frustrations that health care workers witnessed daily. For example, by sharing the experience of patients saying their last words to loved ones through iPad screens, frontline providers were able to provide a window into the emotional toll of the pandemic. Additionally, the amplifier @IMPACT4HC was able to raise awareness about insufficient personal protective equipment [12].

Lastly, amplifiers can provide a private space for the community, fostering social relationships where professionals can privately share personal and professional news and updates. Having a safe space out of the public discourse to “break plates” with others going through similar professional experiences can be both validating and cathartic. The COVID-19 pandemic, particularly the initial waves, politicization, and public divisiveness, is a unique and often extreme stressor for many health professionals; sharing experiences with peers and colleagues and discovering and discussing the similarities in our experiences was described as therapeutic by a number of our members. Additionally, advocacy on social media is often met with harassment, and the amplifier can likewise serve as a community of colleagues who are going through similar experiences [13]. As an example, one of the coauthors (MDR) was the target of xenophobic and racist attacks after directing remarks in Spanish during a press conference with Illinois government officials highlighting the disproportionate burden of COVID-19 among Latino people. This private space not only provided emotional support but also advice on how to respond to online harassment.

Subspecialty amplifiers created during the pandemic allowed for rapid collaboration and dissemination of specialty-specific medical information, something that was necessary, especially at the start of the pandemic. For example, one of the authors (SJ) created a Women in Medicine amplifier focused on amplifying the work and successes of women across specialties, providing a support network for women in the community who may be harassed online or in person, and a way to share challenges and struggles in a supportive environment. Another author (EB) developed an endocrinology amplifier, which allowed for communication regarding novel presentations of endocrine disorders such as profound hyperglycemia and diabetic ketoacidosis in patients with COVID-19. More broadly, this amplifier allowed endocrinologists to communicate in real time and share information, offer peer support, and amplify one another’s work and research. Additionally, when national conferences became virtual, the amplifier was used to promote and support trainee work such as abstracts and oral presentations that otherwise would not have received appropriate notice due to the virtual platform [14].

## Combating Misinformation and Disinformation

The widespread dissemination of misleading or false medical and public health information poses a serious threat to health and safety, especially in a pandemic [15]. This can be misinformation (ie, the spreading of false or inaccurate information) or disinformation (ie, spreading misinformation with the intent to deceive) [16]. Social media platforms have become a major source of health information for laypeople [11]. This democratization of health information has numerous benefits and was useful during a pandemic where information rapidly evolved and guidelines were updated as new evidence emerged; however, content related to health and medicine distributed through social media channels is largely unregulated by private platforms. In March 2020 alone, there were 550 million tweets that included the terms “coronavirus,” “covid19,” or “pandemic,” none of which were fact-checked via official mechanisms [17]. Wu and McCormick [15] argue that it is an ethical imperative and professional obligation for physicians to address false or misleading health information on the internet. Many health care professionals feel obligated to undertake efforts to directly combat disinformation, either on a personal level or at the behest of their professional organizations [18,19]. The American Academy of Pediatrics, for example, has provided its members with resources to communicate key messages and combat misinformation [20]; it also provides members with courses and other resources to communicate key messages with families. Because social media is a major platform for circulating misinformation or disinformation [21], it is also a key place to address or correct it [22].

Amplifiers, such as the IMPACT amplifier on Twitter, help communicate potential messaging of responses (ie, comments, quote tweets) in real time, as well as sharing those messages more widely. They also allow amplifier members a place to discuss among themselves disinformation in need of additional follow-up. For example, within the IMPACT amplifier, a member might share a piece of disinformation that they feel could be better addressed with more nuance in an infographic or a video. This can then be escalated to the team within the coalition developing Myth Buster infographics [23], following the Fact, Myth, Fallacy, Fact refutation strategy for combating misinformation, popularized by climate change activists and described in *The Debunking Handbook* [24]. Other groups such as No License For Disinformation use their own amplifier to facilitate group discussion, identify misinformation, and alert the public and platforms that content is incorrect [25].

## Responding to Harassment

A survey study carried out by some of the authors prior to the COVID-19 pandemic found that 1 in 4 physicians reported being personally attacked on social media and 1 in 6 female physicians reported being sexually harassed on social media [13,26]. Online harassment of physicians has likely worsened during the COVID-19 pandemic [27]. In a study published in *Nature*, almost 60% of responding scientists who had commented on COVID-19 to the media or posted on social media said they had experienced attacks on their credibility, and 15% said they had received death threats [28]. Individuals who experience harassment both online and offline report emotional distress and fear. This emotional distress may contribute to moral injury or burnout in physicians who use social media, compounding the effect of the widespread burnout affecting health care professionals during the COVID-19 pandemic [29,30]. Amplifiers may mitigate the professional impact and emotional distress experienced by physicians who are harassed on social media. If a physician belongs to an amplifier, their initial immediate response to harassment can be to share the offending posts with amplifier members. The amplifier provides a semiprivate space for the harassed physician to plan their response or debrief with colleagues. The amplifier can also become a way to protect one another as members of the group are alerted when another member is attacked or targeted by those attempting to spread disinformation. When an individual finds themselves a target, other members of the amplifier respond with data and evidence in the hopes of drowning out bad information with good information, or all members of the amplifier report the offending social media account [27]. For example, when one of our members became the subject of harassment by a radio station account for not working seriously because they were working from home, another member alerted the amplifier members to start responding with stories of burnout, which generated a large number of replies from health care workers about why they were burned out [31].

## Future Directions

Our future directions include expanding beyond COVID-19 to other public health topics, such as gun violence and reproductive justice. We recently partnered with AFFIRM, a 501(c)(3) nonprofit organization, to sponsor a Chicago-based discussion on gun violence with a panel of speakers including our chief diversity inclusion officer and cofounder. Our director of community engagement is now crowdsourcing a list of health care workers interested in promoting and advocating for the prevention of gun violence [32]. In addition, we have formalized an internship program with the University of Chicago to have a summer-funded intern to continue to produce infographics to share on social media. Lastly, we have received recognition and a grant from the Association of American Medical Colleges, which provides resources for us to continue to teach trainees how to combat misinformation on social media [33].

## Conclusion

Drawing lessons from this experience, it is imperative for medical professionals to utilize all available tools to disseminate accurate medical information and combat disinformation while minimizing harm related to personal and professional harassment that can come with social media advocacy. Understanding the successful use of health professional amplifiers is a substantial way that physicians and other public health professionals can achieve this essential goal.

## Abbreviations

IMPACT: Illinois Medical Professional Action Collaborative Team
